# Effects of the Glycerol/Quercetin Ratio on the Structure and Tensile Behavior of PVA-Based Films

**DOI:** 10.3390/polym18192452

**Published:** 2026-10-08

**Authors:** Sarah Kalli Silva da Silva, Nathalia Vieira Villar de Nunes, Chiara das Dores do Nascimento, Eduarda Vieira Silva, Everton Granemann Souza, Camila Monteiro Cholant, Ivandra Ignes de Santi, Amanda Dantas de Oliveira, Cesar Aguzzoli, Alexandre Ferreira Galio, André Luiz Missio

**Affiliations:** 1 Graduate Program in Materials Science and Engineering (PPGCEM), Federal University of Pelotas (UFPel), Pelotas 96010-610, RS, Brazil; sarah.silva@ufpel.edu.br (S.K.S.d.S.); nathalia.vieira@ufpel.edu.br (N.V.V.d.N.); chiaradnascimento@gmail.com (C.d.D.d.N.); eduarda.vieira@ufpel.edu.br (E.V.S.); camila.scholant@gmail.com (C.M.C.); ivandrasanti9@gmail.com (I.I.d.S.); amandaoliveira82@gmail.com (A.D.d.O.); 2 Graduate Program in Sciences and Technologies in Education (PPGCITED), Federal Institute of Education, Science and Technology of Rio Grande do Sul (IFSul), Pelotas 96060-290, RS, Brazil; 3 Graduate Program in Electronics and Computer Engineering (MEEC), Catholic University of Pelotas (UCPel), Pelotas 96015-560, RS, Brazil; 4 Graduate Program in Materials Engineering and Science (PPGMAT), University of Caxias do Sul (UCS), Caxias do Sul 95070-560, RS, Brazil; caguzzol@ucs.br; 5 Graduate Program in Materials Science and Engineering (PPCEM), Federal University of Pampa (UNIPAMPA), Bagé 96413-172, RS, Brazil; alexandregalio@unipampa.edu.br

**Keywords:** poly(vinyl alcohol), glycerol, quercetin, active films, antioxidant activity, tensile properties

## Abstract

Poly(vinyl alcohol) (PVA)-based films containing glycerol and quercetin were prepared by casting as potential active-packaging materials. The effects of the glycerol/quercetin ratio on structural, thermal, mechanical, barrier, and antioxidant properties were investigated. Neat PVA and ternary formulations with PVA/glycerol/quercetin ratios of 70/20/10 (PVA+Gly+Q10) and 70/10/20 (PVA+Gly+Q20) by mass were characterized by FTIR, XRD, SEM, contact angle, TGA/DTG, tensile testing, water vapor permeability (WVP), total phenolic content, and DPPH assays. FTIR indicated changes in hydrogen bonding without new covalent functionalities, while apparent whole-film crystallinity indices were 16.2%, 11.1%, and 10.6%, respectively. Tensile tests included additional glycerol-rich and quercetin-rich endpoint controls to characterize the mechanical range of the formulation series. Neat PVA exhibited the highest maximum stress (15.67±1.49 MPa). PVA+Gly+Q20 showed a strain at break of 256.87±115.68% and an apparent tensile modulus of 26.61±3.40 MPa. The glycerol-rich endpoint exhibited high extensibility, whereas the quercetin-rich endpoint showed a markedly higher apparent tensile modulus (404.69±45.72 MPa). DPPH activity increased from 11.18% for PVA to 35.38% and 44.31% for PVA+Gly+Q10 and PVA+Gly+Q20, respectively. PVA+Gly+Q20 also exhibited approximately 68% lower mean WVP than PVA+Gly+Q10, with no significant formulation effect. These results support further development of PVA+Gly+Q20 for active packaging.

## 1. Introduction

The development of functional polymeric films with improved environmental profiles has gained increasing attention in response to the persistence of commodity plastics such as polyethylene (PE) and polyethylene terephthalate (PET). In this context, water-processable polymer matrices capable of incorporating bioactive compounds provide a promising platform for producing materials that can scavenge free radicals, delay oxidative processes, and contribute to food preservation [1,2,3,4,5,6].

Poly(vinyl alcohol) (PVA) is particularly attractive for these systems because of its excellent film-forming ability, high transparency, and recognized biocompatibility [7,8,9]. Its hydroxyl-rich structure promotes extensive intermolecular hydrogen bonding, producing cohesive films but also restricting chain mobility and contributing to intrinsic brittleness [10]. Glycerol is therefore commonly incorporated as a plasticizer to increase free volume, flexibility, and processability [11]. However, its effect is strongly concentration-dependent because increasing glycerol content can also modify water affinity, hydrogen-bonding organization, crystallinity, and thermal degradation pathways within the PVA matrix [12,13,14].

For active packaging applications, improving film flexibility alone is insufficient: the material must also protect oxidation-sensitive products while retaining adequate mechanical integrity, moisture response, and thermal stability during processing and use. Oxidative reactions can promote lipid rancidity, pigment and nutrient degradation, off-flavor formation, and loss of food quality. Incorporating antioxidants into the polymer matrix can localize radical-scavenging activity at the package–product interface and help reduce oxidative deterioration [3,4,15]. Quercetin (3,3^′^,4^′^,5,7-pentahydroxyflavone) is particularly relevant for this purpose because its conjugated aromatic structure and multiple phenolic hydroxyl groups provide efficient reactive oxygen species scavenging [16,17,18]. These functional groups can also establish intermolecular interactions with PVA and glycerol, allowing quercetin to modify molecular packing, chain mobility, crystallinity, wettability, and mechanical behavior while imparting antioxidant functionality [19,20,21,22].

Previous studies have explored binary PVA/quercetin films without glycerol [19], starch/PVA/glycerol matrices for copper sensing [23], and glycerol-containing PVA hydrogel films incorporating quercetin, with and without silver nanoparticles, in which swelling, quercetin release, silver release from AgNP-containing films, and antibacterial effects were evaluated [24]. Taken together, these studies illustrate the use of quercetin and/or glycerol in PVA-based film systems under different application contexts, but leave open a formulation question relevant to active packaging: how does replacing part of the glycerol with quercetin affect the balance between antioxidant activity, water-vapor transport, and mechanical integrity? Addressing this question in a matrix without a second polymer or inorganic phase [25] allows the glycerol/quercetin balance to be examined at a constant PVA content.

This study therefore investigated PVA/glycerol/quercetin films with different glycerol-to-quercetin ratios, relating their structural, morphological, thermal, and wetting characteristics to tensile behavior, water-vapor permeability, extractable phenolic content, and antioxidant activity. It was hypothesized that partial replacement of glycerol by quercetin would shift the balance between plasticization and intermolecular association, modifying strength and extensibility while enhancing antioxidant activity. The tensile analysis was further strengthened by including endpoint formulations spanning the glycerol-to-quercetin substitution range. The aim was to identify a formulation that combines antioxidant functionality with mechanical and water-vapor barrier properties suitable for further active-packaging development.

## 2. Materials and Methods

### 2.1. Materials

Poly(vinyl alcohol) (PVA; CAS No. 9002-89-5), synthesis grade, containing ≥98% poly(vinyl alcohol), was purchased from Dinâmica Química Contemporânea Ltda. (Indaiatuba, SP, Brazil). Quercetin dihydrate ($C_{15}H_{10}O_{7}\cdot 2H_{2}O$; CAS No. 6151-25-3) was purchased from Inlab Confiança (São Paulo, SP, Brazil). Glycerol ($C_{3}H_{8}O_{3}$; CAS No. 56-81-5), analytical grade, was purchased from Dinâmica Química Contemporânea Ltda. (Indaiatuba, SP, Brazil). All reagents were used as received without further purification.

### 2.2. Film Preparation

PVA-based films containing glycerol and quercetin were prepared by solvent casting, following a procedure previously employed by the research group, with modifications [26]. Three formulations were investigated: a neat PVA control and two PVA/glycerol/quercetin formulations, as summarized in Table 1. The compositions were calculated on a total dry-mass basis, excluding the distilled water used as the processing solvent.

All films were prepared at a nominal dry grammage of 100 g m^−2^, defined by the dry mass added per unit casting area rather than by a gravimetric measurement after drying. For the circular casting plates with a diameter of 100 mm and an effective area of 7.854 × 10^−3^ m^2^, this corresponded to a total dry mass of 0.7854 g per film. The mass of each component was then calculated according to its respective dry-mass fraction, as presented in Table 1.

For each formulation, the required amount of PVA was gradually added to a beaker containing 30 mL of distilled water. The mixture was maintained under magnetic stirring at 70 °C for 60 min to ensure complete dissolution of the polymer. Glycerol and quercetin were subsequently added in the proportions specified in Table 1, and stirring was continued at the same temperature for an additional 5 min to incorporate the additives into the PVA solution prior to casting. Under these conditions, quercetin remained dispersed rather than fully dissolved in the aqueous medium. The 5-min interval was used as a standardized processing step to distribute the additives immediately before casting and was not intended to establish complete quercetin dissolution or equilibrium dispersion. Because particle-size distribution and sedimentation kinetics were not quantified, whether a longer dispersion period would further alter the homogeneity of the film-forming dispersion cannot be determined from the present data. Accordingly, the resulting mixtures are referred to as film-forming dispersions.

The resulting film-forming dispersion was then poured into a 100-mm-diameter glass casting plate placed on a leveled surface. The samples were dried in a forced-air circulation oven at 40 °C for 24 h. After drying, the films were carefully peeled from the casting plates and stored in a desiccator until characterization.

### 2.3. Film Characterization

#### 2.3.1. Fourier Transform Infrared Spectroscopy (FTIR)

Attenuated total reflectance Fourier transform infrared spectroscopy (ATR-FTIR) was used to characterize the functional groups and assess spectral changes associated with interactions among PVA, glycerol, and quercetin. The spectra were recorded using a Spectrum 400 spectrometer (PerkinElmer, Waltham, MA, USA) equipped with an ATR accessory containing a diamond crystal. For each sample, 30 scans were accumulated over the range 4000–900 cm^−1^ at a spectral resolution of 4 cm^−1^. The film surface was placed directly in contact with the ATR crystal, and similar contact-pressure conditions were maintained during all acquisitions. The spectra were baseline-corrected and normalized to facilitate comparison among the formulations. A constant vertical offset was applied exclusively for graphical presentation and did not alter the positions of the absorption bands. The instrumental procedure was based on that reported by Nunes et al. [27].

#### 2.3.2. X-Ray Diffraction

The crystalline structure of the films was evaluated by X-ray diffraction (XRD) using a MiniFlex 600 diffractometer (Rigaku, Tokyo, Japan) operated with Cu Kα radiation (λ=0.15406 nm) at 40 kV and 15 mA. The diffraction patterns were recorded in continuous-scan mode over a 2θ range of $10^{\circ }$–$60^{\circ }$ at a scan rate of $2^{\circ }\;\min^{-1}$ and a data interval of $0.02^{\circ }$. After baseline correction, the diffraction profiles were deconvoluted into three narrower ordered contributions (C1–C3) and two broad diffuse contributions (A1 and A2) using Gaussian functions in OriginPro 2023 (Student Version; OriginLab Corporation, Northampton, MA, USA) [28]. A globally constrained fitting procedure was applied, with the positions and widths of C1–C3 shared across the three formulations, while their intensities and the parameters of A1 and A2 were optimized independently for each profile.

The apparent crystallinity index, $X_{c}$, of the films was calculated from the integrated areas of the fitted ordered and diffuse contributions as

(1) $$X_{c}\;\left(\%\right)=\frac{A_{c}}{A_{c}+A_{a}}\times 100,$$

where $A_{c}$ is the summed integrated area of the ordered contributions C1–C3 and $A_{a}$ is the summed integrated area of the broad diffuse contributions A1 and A2. Because this index is normalized to the total fitted diffraction area of each film, it represents an apparent whole-film crystallinity index and is not corrected for the PVA mass fraction. Accordingly, comparisons between neat PVA and the additive-containing formulations should not be interpreted as direct measurements of changes in the intrinsic crystallinity of the PVA fraction.

#### 2.3.3. Scanning Electron Microscopy

The surface morphology of the films was examined by scanning electron microscopy (SEM) using a VEGA3 microscope (TESCAN, Brno, Czech Republic). Prior to imaging, the specimens were mounted on aluminum stubs using conductive carbon tape and sputter-coated with gold to improve surface conductivity and minimize charging effects. The micrographs were acquired using a secondary-electron detector at a working distance of approximately 11 mm and nominal magnifications of 100×, 500×, and 1000×. The PVA film was examined at an accelerating voltage of 15 kV, whereas the PVA+Gly+Q10 and PVA+Gly+Q20 films were examined at 10 kV, in accordance with the acquisition parameters recorded in the original micrographs.

#### 2.3.4. Time-Dependent Apparent Water Contact Angle

Film wettability was evaluated by the sessile-drop method using a Theta Lite TL100 optical tensiometer (Biolin Scientific, Gothenburg, Sweden) controlled by the OneAttension software, version 4.0. Measurements were performed on the air-exposed surface of the films, corresponding to the surface that remained exposed to air during film drying.

For each measurement, a 10μL droplet of distilled water was gently deposited onto the film surface. The evolution of the droplet profile was recorded for 60 s at an acquisition rate of 20 frames per second. The apparent water contact angle was automatically calculated by the software from the droplet profile using the same fitting model for all formulations.

One droplet was deposited on each of three independently prepared films for every formulation. Each film was treated as an independent experimental unit (n=3). The apparent water contact angles were determined at 1, 10, 20, 30, 40, 50, and 60 s and expressed as mean ± standard deviation. Because droplet spreading and water absorption occurred during the measurements, the results were interpreted as time-dependent apparent water contact angles rather than equilibrium contact angles.

#### 2.3.5. Thermogravimetric Analysis

The thermal behavior of the films was evaluated using a TGA-50 thermogravimetric analyzer (Shimadzu Corporation, Kyoto, Japan). Approximately 4 mg of each film was placed in a platinum crucible and heated from 28 to 700 °C at 10 °C min^−1^ under nitrogen flowing at 50 mL min^−1^. One measurement was performed for each formulation.

The mass was normalized to the initial sample mass and expressed as the percentage of remaining mass. Derivative thermogravimetric (DTG) curves were calculated numerically in OriginPro [28] as $d[m\left(T\right)/m_{0}\times 100]/dT$, where m(T) is the sample mass at temperature *T* and $m_{0}$ is the initial sample mass. Although measurements were conducted up to 700 °C, the curves are presented up to 650 °C to emphasize the relevant degradation events.

The characteristic parameters $T_{5\%}$ and $T_{10\%}$ correspond to the temperatures at 5% and 10% mass loss, respectively. The extrapolated onset temperature ($T_{\mathrm{onset}}$) of the principal degradation step was determined from the intersection between the tangent at the inflection region of the corresponding TGA mass-loss step and the preceding baseline. $T_{\max,\mathrm{DTG}}$ corresponds to the temperature of the minimum of the principal DTG peak, and the residual mass ($R_{650}$) was determined at 650 °C. Because one measurement was obtained for each formulation, these thermal parameters were interpreted descriptively.

#### 2.3.6. Tensile Testing

Mechanical properties were determined using a universal testing machine (DL2000, EMIC, São José dos Pinhais, PR, Brazil) equipped with a 1-kN load cell, following a procedure adapted from ASTM D882-18 [29]. For each formulation, five tensile specimens were tested (n=5); each specimen corresponded to an independently prepared film sample. All films were prepared by the casting procedure described in Section 2.2 and dried in a forced-air oven at 40 °C for 24 h. Before testing, the specimens were conditioned for 48 h at 22 °C and 50±5% relative humidity. Tensile tests were subsequently performed at 22 °C and 50±5% relative humidity.

The mean specimen thicknesses for PVA were 0.14, 0.15, 0.16, 0.17, and 0.17 mm, whereas those for PVA+Gly+Q10 were 0.12, 0.12, 0.14, 0.15, and 0.15 mm and those for PVA+Gly+Q20 were 0.11, 0.12, 0.12, 0.14, and 0.15 mm. Two additional endpoint formulations were prepared using the same casting procedure exclusively for the tensile analysis: PVA+Gly30 (70/30/0 PVA/glycerol/quercetin) and PVA+Q30 (70/0/30). Their mean specimen thicknesses were 0.15, 0.15, 0.16, 0.16, and 0.16 mm for PVA+Gly30 and 0.15, 0.15, 0.16, 0.17, and 0.18 mm for PVA+Q30.

The films were cut into rectangular strips approximately 90 mm long and 20 mm wide. For each specimen, width and thickness were measured at three positions along the gauge region using a digital caliper and a digital micrometer, respectively. The corresponding mean width and thickness were used to calculate the initial cross-sectional area for the engineering-stress calculation.

The initial grip separation was 50 mm, and the crosshead speed was 12 mm min^−1^. The specimens were secured using flat-faced film grips with rubber-coated jaws to distribute the clamping pressure and minimize slippage or premature damage. Tests in which the specimen slipped from the grips or fractured outside the gauge region were discarded.

Engineering stress (σ) was calculated as

(2) $$\sigma =\frac{F}{wt},$$

where *F* is the applied force, *w* is the mean initial specimen width, and *t* is the mean specimen thickness measured along the gauge region. Tensile strength was defined as the maximum engineering stress reached during the test.

Engineering strain (ε) was calculated as

(3) $$\varepsilon =\frac{L-L_{0}}{L_{0}},$$

where $L_{0}$ is the initial grip separation and *L* is the instantaneous distance between the grips. Strain at break ($\varepsilon_{b}$) was defined as the engineering strain at specimen rupture and was expressed as a percentage.

Because strain was obtained from grip displacement rather than local extensometry, stiffness is reported as an apparent tensile modulus. The initial seating/toe region was excluded from modulus determination. For each specimen, the first stable linear segment in the initial low-strain ascending region was fitted by linear regression, and only fits with $R^{2}\geq 0.99$ were accepted. Toe compensation for modulus determination was performed by extrapolating the fitted segment to zero stress. The apparent tensile modulus was taken as the slope of this fitted segment. Toe compensation was used only for modulus determination and was not used to shift the complete stress–strain curves.

#### 2.3.7. Total Phenolic Content and DPPH Radical-Scavenging Activity

For the antioxidant analyses, film samples were cut into small pieces, and 0.1 g of each film was immersed in 2 mL of ethanol for 24 h at room temperature without agitation, corresponding to an initial film-to-solvent ratio of 50 mg mL^−1^. The extraction procedure was adapted from Luzi et al. [19]. The resulting extracts were used for both total phenolic content and DPPH analyses.

Total phenolic content was determined using the Folin–Ciocalteu method, following de Santi et al. [30]. An aliquot of 0.5 mL of each film extract was mixed with 2.5 mL of Folin–Ciocalteu reagent and 5 mL of 20% sodium carbonate solution. The mixtures were incubated at 50 °C for 1 h, and absorbance was measured at 750 nm using a UV–Vis spectrophotometer. Total phenolic content was calculated using the gallic acid calibration equation A=0.0022C+0.2523, where *A* is the absorbance and *C* is the concentration expressed in mg GAE L^−1^. Results were expressed as milligrams of gallic acid equivalents per liter of extract.

The antioxidant activity of the films was evaluated using the 2,2-diphenyl-1-picrylhydrazyl (DPPH) radical-scavenging assay, following Pellati et al. [31], with modifications.

For the assay, 0.1 mL of each film extract was mixed with 3.9 mL of a DPPH solution at a concentration of $6.0\times 10^{-5}$ mol L^−1^. After homogenization, the reaction mixtures were incubated for 60 min at 25 °C, protected from light, consistent with the reaction period employed for PVA-based antioxidant films by Luzi et al. [19].

Absorbance was subsequently measured at 515 nm using a UV–Vis spectrophotometer. Ethanol was used as the instrumental blank. The negative control was prepared by mixing 0.1 mL of ethanol with 3.9 mL of the DPPH solution.

The DPPH radical-scavenging activity was expressed as the percentage of inhibition and calculated according to Equation (4):

(4) $$\mathrm{DPPH}\;\mathrm{inhibition}\;\left(\%\right)=\frac{A_{\mathrm{control}}-A_{\mathrm{sample}}}{A_{\mathrm{control}}}\times 100,$$

where $A_{\mathrm{control}}$ is the absorbance of the negative control containing DPPH without film extract, and $A_{\mathrm{sample}}$ is the absorbance of the reaction mixture containing the corresponding film extract.

For each formulation, three independently prepared film samples were extracted and analyzed for total phenolic content and DPPH radical-scavenging activity. Results were expressed as mean ± standard deviation (n=3).

#### 2.3.8. Water Vapor Permeability

Water vapor permeability (WVP) was determined using an adapted gravimetric water-cup method based on ASTM E96/E96M, accounting for the resistance of the stagnant air layer between the water surface and the film according to McHugh et al. [32] and Gennadios et al. [33].

Polypropylene conical tubes with a nominal capacity of 15 mL were used as permeation cells. Each tube contained 15 mL of distilled water, and the film specimen was clamped between the tube body and a perforated screw cap, covering an exposed area of $2.2698\times 10^{-4}$ m^2^. The initial distance between the water surface and the underside of the film was 8.439 mm. The assemblies were placed in a sealed desiccator containing freshly dried silica gel and maintained at 25 °C. Mass was recorded at 1 h intervals, and the water vapor transmission rate (WVTR) was calculated from the magnitude of the slope of the linear mass-loss region:

(5) $$\mathrm{WVTR}=\frac{\left|dm/dt\right|}{A},$$

where |dm/dt| is the mass-loss rate in g h^−1^ and *A* is the exposed film area in m^2^.

The water vapor partial pressure at the inner film surface, $p_{i}$, was calculated from the diffusion relationship for water vapor through stagnant air:

(6) $$N_{w}=\frac{PD_{\mathrm{wa}}}{RTz}\ln\left(\frac{P-p_{i}}{P-p_{w}}\right),$$

where $N_{w}$ is the molar water vapor flux, *P* is the total pressure, $D_{\mathrm{wa}}$ is the diffusivity of water vapor in air, *R* is the universal gas constant, *T* is the absolute temperature, *z* is the air-gap height, and $p_{w}$ is the saturation water vapor pressure at the water surface. The calculation used P=101,325 Pa, $D_{\mathrm{wa}}=2.6\times 10^{-5}$ m^2^ s^−1^, R=8.314 Pa m^3^ mol^−1^ K^−1^, and T=298.15 K, with *z* expressed in meters. The molar flux was obtained from the measured WVTR using $N_{w}=\mathrm{WVTR}/\left(3600M_{w}\right)$, where $M_{w}=18.015$ g mol^−1^ is the molar mass of water. WVP was calculated from the corrected vapor pressure difference across the film:

(7) $$\mathrm{WVP}=\frac{\mathrm{WVTR}\;L}{p_{i}-p_{o}},$$

where *L* is the mean specimen thickness in meters and $p_{o}$ is the water vapor partial pressure in the desiccator, with both partial pressures expressed in Pa. The equation yields WVP in g m^−1^ h^−1^ Pa^−1^; conversion to the reporting units of g mm m^−2^ h^−1^ kPa^−1^ requires multiplication by $10^{6}$. Measurements were performed in triplicate for each formulation and reported as mean ± standard deviation.

### 2.4. Statistical Analysis

Statistical analyses were performed using OriginPro [28]. For the tensile analysis, each replicate corresponded to one tensile specimen from an independently prepared film sample (n=5 per formulation). Mechanical data were analyzed using Welch’s one-way ANOVA, with formulation as the fixed factor. This approach was selected because it is robust to unequal variances among groups. When a significant formulation effect was detected, Games–Howell multiple-comparison tests were applied separately to maximum stress, strain at break, and apparent tensile modulus. Statistical significance was considered at p<0.05.

WVP data were analyzed using Welch’s one-way ANOVA because of the unequal variances observed among formulations. Total phenolic content of PVA+Gly+Q10 and PVA+Gly+Q20 was compared using Welch’s *t*-test; neat PVA, which yielded no measurable response under the assay conditions, was excluded from this concentration-based comparison. DPPH radical-scavenging data were analyzed using Welch’s one-way ANOVA followed by the Games–Howell multiple-comparison test. Differences were considered significant at p<0.05.

Time-dependent apparent water contact-angle data were reported as mean ± standard deviation and analyzed descriptively. Results obtained from single measurements were also interpreted descriptively.

## 3. Results and Discussion

### 3.1. Molecular Interactions Assessed by FTIR

**Figure 1:**
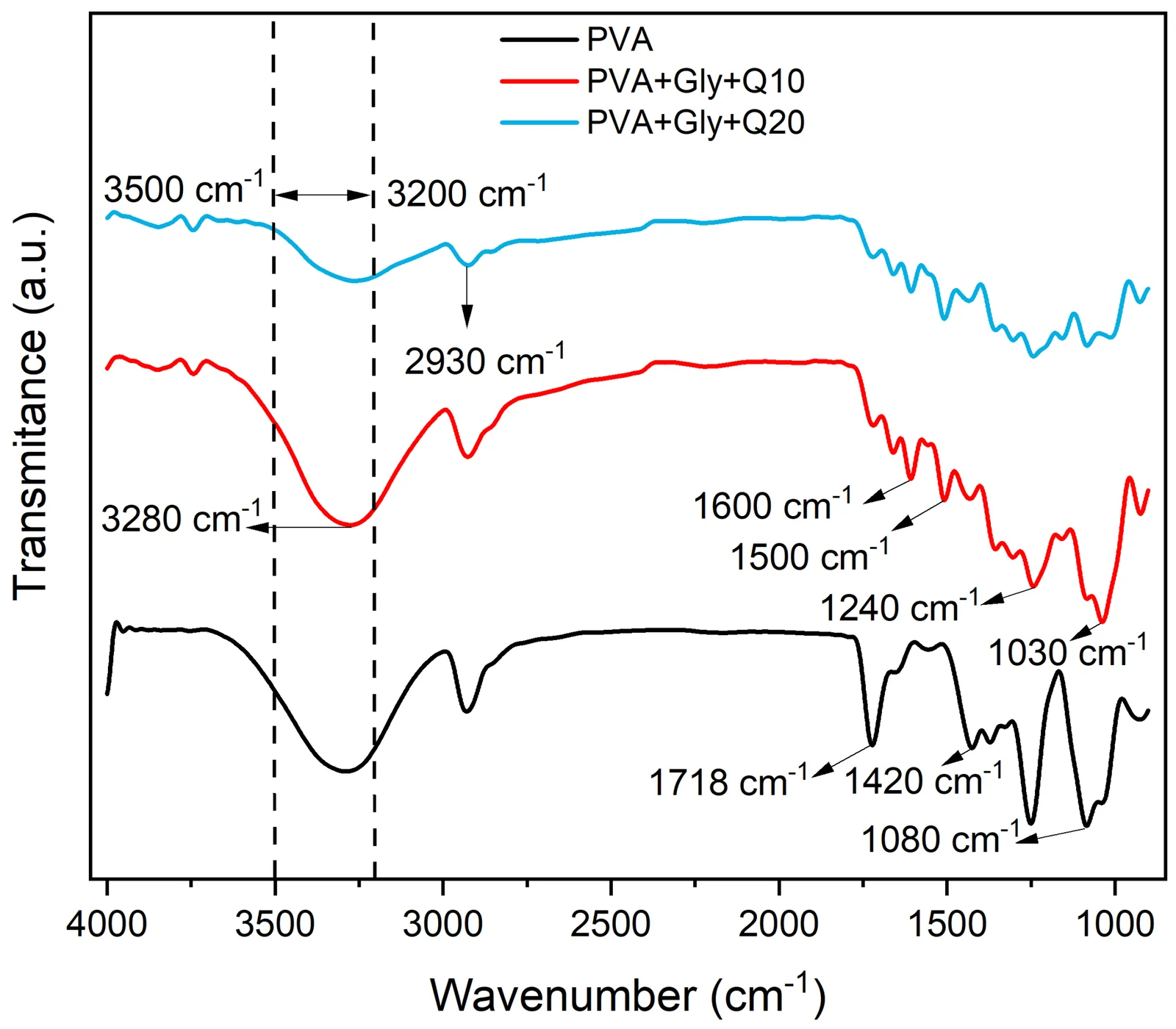
Normalized ATR-FTIR spectra of neat PVA, PVA+Gly+Q10, and PVA+Gly+Q20 films. A constant vertical offset was applied to the spectra for clarity.

The normalized ATR-FTIR spectra of neat PVA and the PVA/glycerol/quercetin films are presented in Figure 1.

Neat PVA exhibited a broad absorption band between 3500 and 3200 cm^−1^, centered at approximately 3280 cm^−1^, assigned to O–H stretching within the hydrogen-bonded polymer network. The band at approximately 2930 cm^−1^ was attributed to aliphatic C–H stretching of the PVA backbone, whereas the absorptions near 1420 and 1080 cm^−1^ were associated with CH_2_ bending and C–O stretching, respectively [13].

An absorption band near 1718 cm^−1^ was observed in the neat-PVA spectrum. Together with the contribution near 1240 cm^−1^, this feature is associated with residual acetate units resulting from incomplete hydrolysis of the poly(vinyl acetate) precursor used in PVA production [34]. Its lower relative prominence in the additive-containing films may be related, at least in part, to the lower PVA fraction in these formulations and to spectral overlap with vibrations from glycerol and quercetin.

Progressive replacement of glycerol by quercetin modified the profile of the broad O–H band. This change may reflect a redistribution of hydrogen bonding among the hydroxyl groups of PVA, glycerol, and quercetin [13,19].

The clearest spectral changes associated with quercetin addition were observed between approximately 1650 and 1500 cm^−1^. Bands near 1600 and 1500 cm^−1^ became more distinguishable in the PVA+Gly+Q10 and PVA+Gly+Q20 spectra and were attributed primarily to aromatic C=C stretching vibrations of quercetin [19]. Additional changes between 1250 and 1000 cm^−1^ arise from overlapping C–O vibrations of PVA, glycerol, and the phenolic groups of quercetin.

The crystallinity-sensitive PVA contribution commonly reported near 1141 cm^−1^ was not sufficiently resolved from the neighboring C–O bands to support quantitative conclusions regarding crystallinity from FTIR alone; this aspect was therefore evaluated by XRD.

No new well-resolved absorption bands indicative of additional covalent functionalities were observed. The spectral changes are therefore more readily attributed to the physical incorporation of glycerol and quercetin and to changes in the hydrogen-bonding environment of the PVA matrix.

### 3.2. Crystalline Structure Assessed by XRD

**Figure 2:**
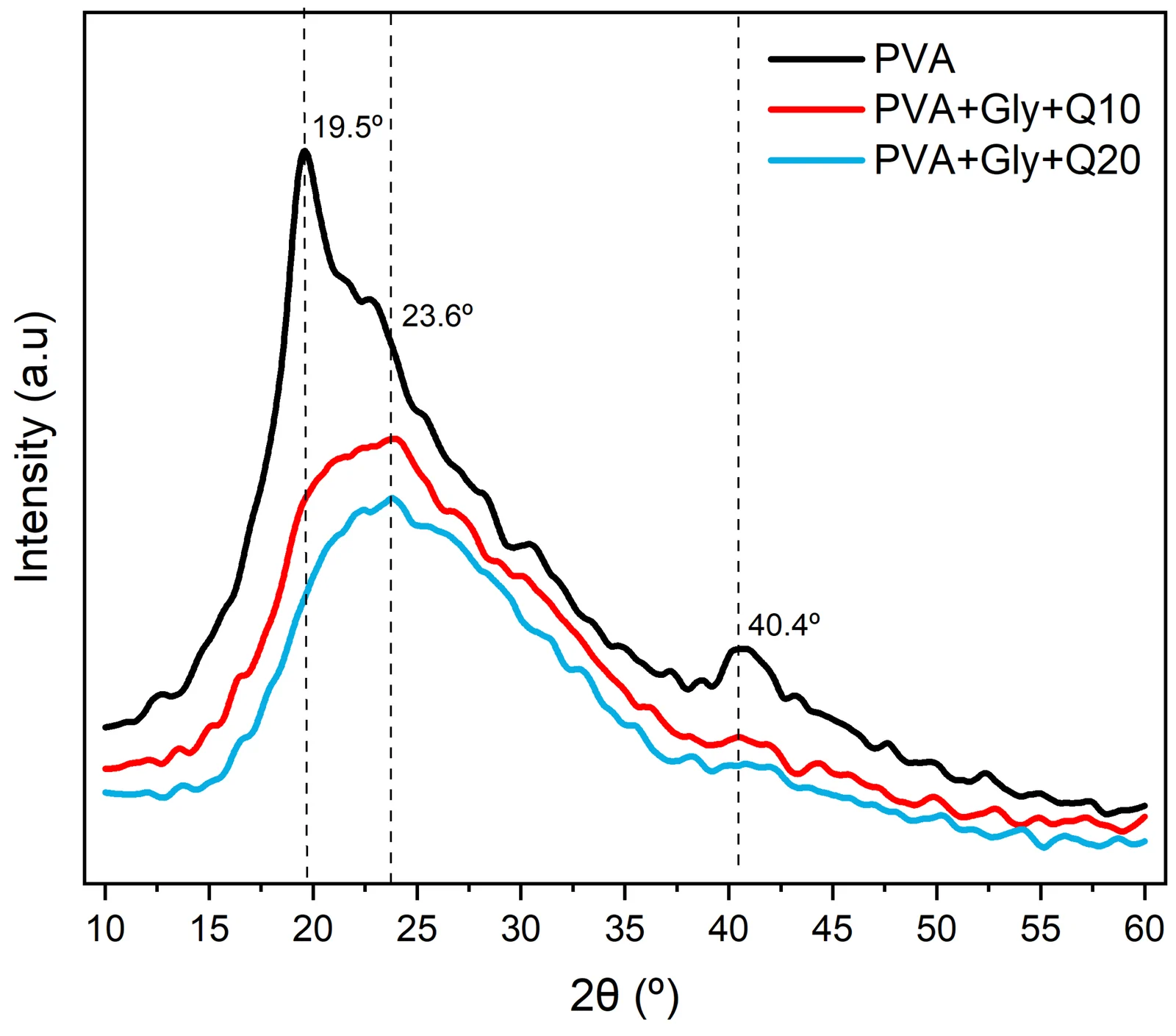
X-ray diffraction patterns of PVA, PVA+Gly+Q10, and PVA+Gly+Q20. Dashed lines indicate the main diffraction features observed at approximately $19.5^{\circ }$, $23.6^{\circ }$, and $40.4^{\circ }$.

The XRD patterns of PVA, PVA+Gly+Q10, and PVA+Gly+Q20 are shown in Figure 2. PVA exhibited the characteristic semicrystalline profile of the polymer, with a dominant diffraction maximum at approximately $2\theta =19.5^{\circ }$, a broad shoulder in the 22–$25^{\circ }$ region, and a weak broad feature around 40–$41^{\circ }$. Diffraction features close to 19–$20^{\circ }$ and 40–$42^{\circ }$ are commonly reported for semicrystalline PVA [13].

The incorporation of glycerol and quercetin broadened the diffraction profile of PVA. In both additive-containing formulations, the main feature near $19.5^{\circ }$ became less distinct and a broader diffraction envelope was observed between approximately $18^{\circ }$ and $27^{\circ }$. The weak contribution around 40–$41^{\circ }$ also became less defined. These changes are consistent with a reduction in the structural order of the PVA matrix, while the persistence of the main PVA diffraction features at similar angular positions indicates that the remaining ordered regions retained PVA-like packing.

No additional sharp reflections were observed in PVA+Gly+Q10 or PVA+Gly+Q20. Therefore, no separately detectable crystalline phase attributable to quercetin was identified under the measurement conditions, indicating that the incorporated quercetin did not develop sufficient long-range order to generate distinct diffraction reflections.

The diffraction profiles were further analyzed using the globally constrained deconvolution procedure described in Section 2.3.2. The apparent crystallinity index, $X_{c}$, was 16.2% for PVA, 11.1% for PVA+Gly+Q10, and 10.6% for PVA+Gly+Q20, with corresponding sensitivity ranges of 15.7–16.7%, 10.2–12.0%, and 9.8–11.4%, respectively. The fits yielded $R^{2}$ values between 0.9972 and 0.9976 and RMSE values between 2.13 and 3.32 a.u. Detailed fitted profiles, residuals, deconvolution parameters, and sensitivity results are provided in Supplementary Figure S1 and Table S1.

The lower apparent crystallinity indices of the additive-containing films relative to neat PVA are partly influenced by their lower PVA fraction (70 wt.%), since $X_{c}$ was calculated from the diffraction profile of the whole film. At the same time, the broadening and reduced definition of the characteristic PVA reflections indicate changes in the structural organization of the matrix after incorporation of glycerol and quercetin. PVA+Gly+Q10 and PVA+Gly+Q20 showed comparable apparent crystallinity, as indicated by their close values and overlapping sensitivity ranges. These findings complement the FTIR evidence of changes in the hydrogen-bonding environment of the PVA matrix.

### 3.3. Surface Morphology Assessed by SEM

**Figure 3:**
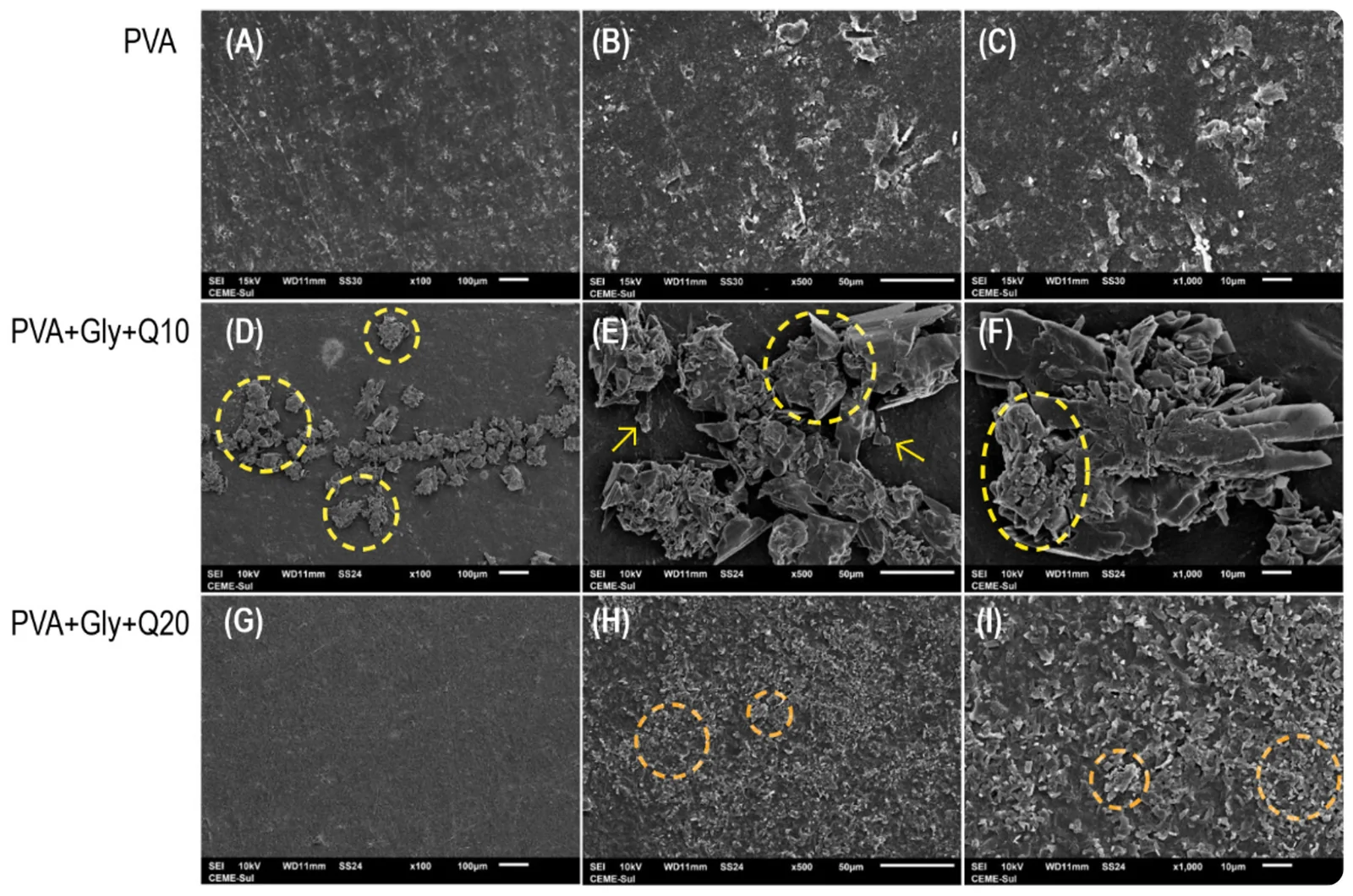
Representative SEM micrographs of the surfaces of PVA (**A**–**C**), PVA+Gly+Q10 (**D**–**F**), and PVA+Gly+Q20 (**G**–**I**) at magnifications of 100× (**A**,**D**,**G**), 500× (**B**,**E**,**H**), and 1000× (**C**,**F**,**I**). Dashed circles and arrows indicate representative surface heterogeneities.

The surface morphologies of PVA, PVA+Gly+Q10, and PVA+Gly+Q20 were examined at different magnifications, as shown in Figure 3. PVA exhibited a continuous surface without large cracks or isolated aggregates at low magnification (Figure 3A). At higher magnifications, fine irregular features were distributed across the surface, producing a moderately rough texture while preserving the continuity of the polymer matrix (Figure 3B,C).

The morphology of PVA+Gly+Q10 differed markedly from that of PVA. At low magnification, coarse and spatially localized aggregates extending over several tens of micrometers were observed on an otherwise comparatively smooth matrix (Figure 3D). Higher-magnification images revealed irregular, plate-like, and fragmented structures with pronounced topographical discontinuities between these deposits and the surrounding matrix (Figure 3E,F). This morphology indicates a heterogeneous surface distribution of the film components. Similar morphological heterogeneities have also been reported in PVA/quercetin films by Luzi et al. [19].

PVA+Gly+Q20 exhibited a different surface distribution. At low magnification, the film appeared comparatively continuous and lacked the large isolated aggregates observed in PVA+Gly+Q10 (Figure 3G). At 500× and 1000×, however, a dense granular texture composed of fine irregular domains was distributed across the surface, together with a limited number of localized clusters (Figure 3H,I). Thus, PVA+Gly+Q20 was more uniform than PVA+Gly+Q10 at the scale of tens to hundreds of micrometers, while displaying a more finely textured surface at the micrometer scale. The change in the glycerol/quercetin ratio therefore affected the spatial distribution and characteristic size of the observed surface heterogeneities.

The surface heterogeneities observed by SEM do not conflict with the absence of additional sharp reflections in the XRD patterns, since localized domains may be insufficiently ordered or abundant to generate a separately detectable crystalline phase. Taken together, the results show that glycerol and quercetin modified the surface organization of the PVA matrix, whereas XRD demonstrated a reduction in apparent crystallinity relative to neat PVA without supporting a meaningful difference in crystallinity between PVA+Gly+Q10 and PVA+Gly+Q20. The SEM observations are restricted to surface topography and spatial distribution and do not establish the chemical identity of the individual domains or their distribution throughout the film thickness.

### 3.4. Time-Dependent Surface Wettability

**Figure 4:**
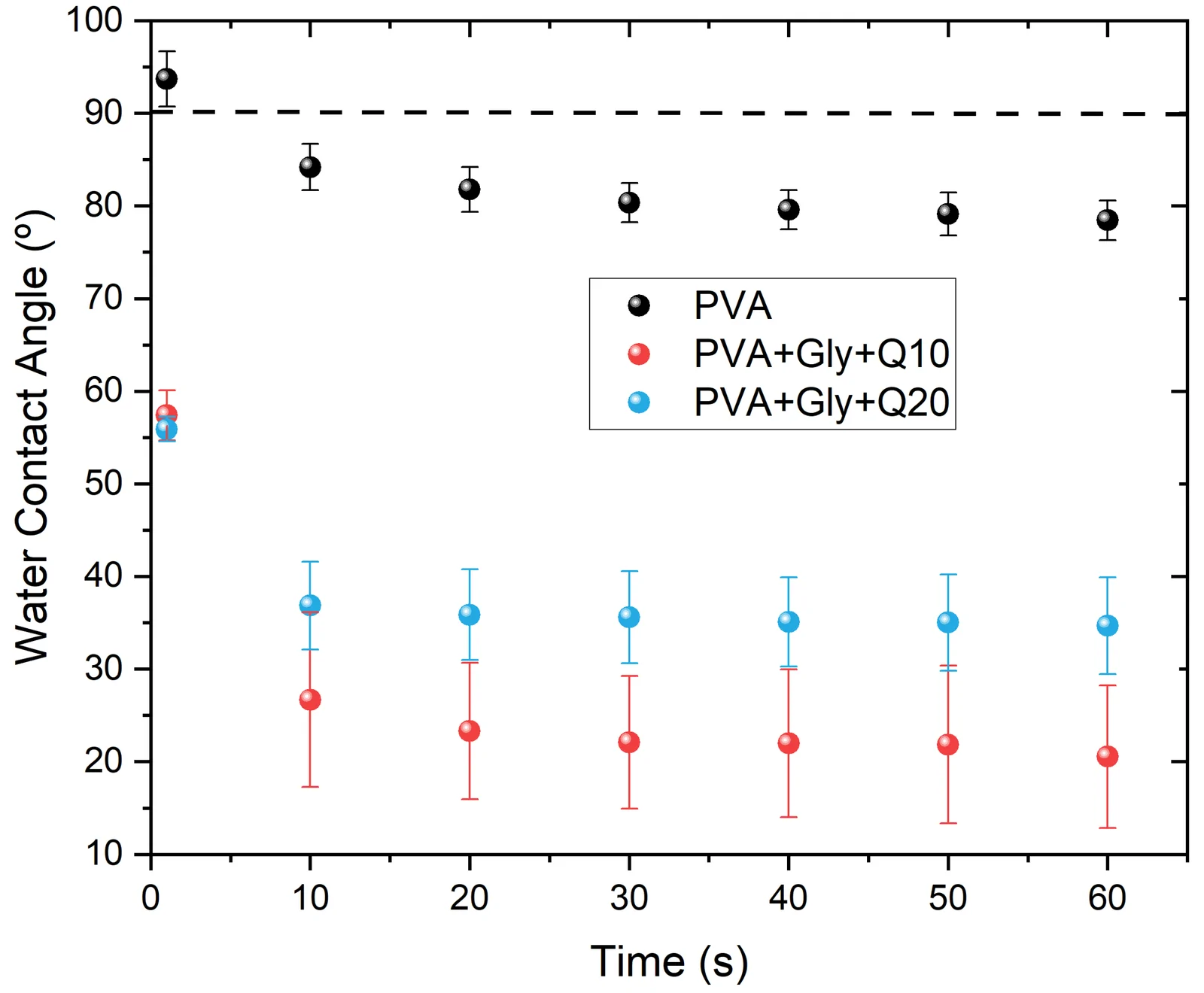
Time-dependent apparent water contact angles of PVA, PVA+Gly+Q10, and PVA+Gly+Q20 over 60 s. Values are expressed as mean ± standard deviation. The horizontal dashed line marks the conventional $90^{\circ }$ boundary between hydrophilic and hydrophobic wetting.

The time-dependent water contact angles of PVA, PVA+Gly+Q10, and PVA+Gly+Q20 are presented in Figure 4. At the initial measurement, PVA exhibited an apparent contact angle of approximately $94^{\circ }$, whereas PVA+Gly+Q10 and PVA+Gly+Q20 showed lower values of approximately $58^{\circ }$ and $56^{\circ }$, respectively.

All films exhibited their largest contact-angle decrease during the first 10 s. For PVA, the contact angle decreased from approximately $94^{\circ }$ to $84^{\circ }$ and then declined more gradually to approximately $78^{\circ }$ at 60 s. Thus, the initially measured value above $90^{\circ }$ was transient. This time-dependent behavior is characteristic of water-responsive PVA surfaces, where spreading and water uptake occur during droplet contact. Majerczak et al. [35] similarly showed that the wetting kinetics of PVA films depend on composition, plasticizer content, and polymer structure, with glycerol promoting faster surface spreading.

PVA+Gly+Q10 exhibited the largest decrease in mean contact angle, from approximately $58^{\circ }$ at the initial measurement to $27^{\circ }$ at 10 s and approximately $20^{\circ }$ after 60 s. PVA+Gly+Q20 also showed rapid initial wetting, decreasing from approximately $56^{\circ }$ to $37^{\circ }$ at 10 s and subsequently remaining close to $35^{\circ }$. The apparent initial wetting rates calculated between 1 and 10 s were 1.05, 3.41, and 2.12° s^−1^ for PVA, PVA+Gly+Q10, and PVA+Gly+Q20, respectively. Although the mean initial rate was higher for PVA+Gly+Q10, the dispersion ranges of the two additive-containing films overlapped during the first 10 s. Their wetting responses became more clearly separated from 20 s onward, with PVA+Gly+Q10 maintaining lower contact-angle values throughout the remainder of the measurement.

The distinct wetting responses are consistent with the different glycerol/quercetin ratios of the two films. PVA+Gly+Q10, which contained the greater glycerol fraction, showed the largest contact-angle decrease, whereas partial replacement of glycerol by quercetin in PVA+Gly+Q20 was accompanied by less extensive spreading. Because both additives varied simultaneously, however, their individual contributions cannot be distinguished from these data.

### 3.5. Thermal-Degradation Behavior

**Figure 5:**
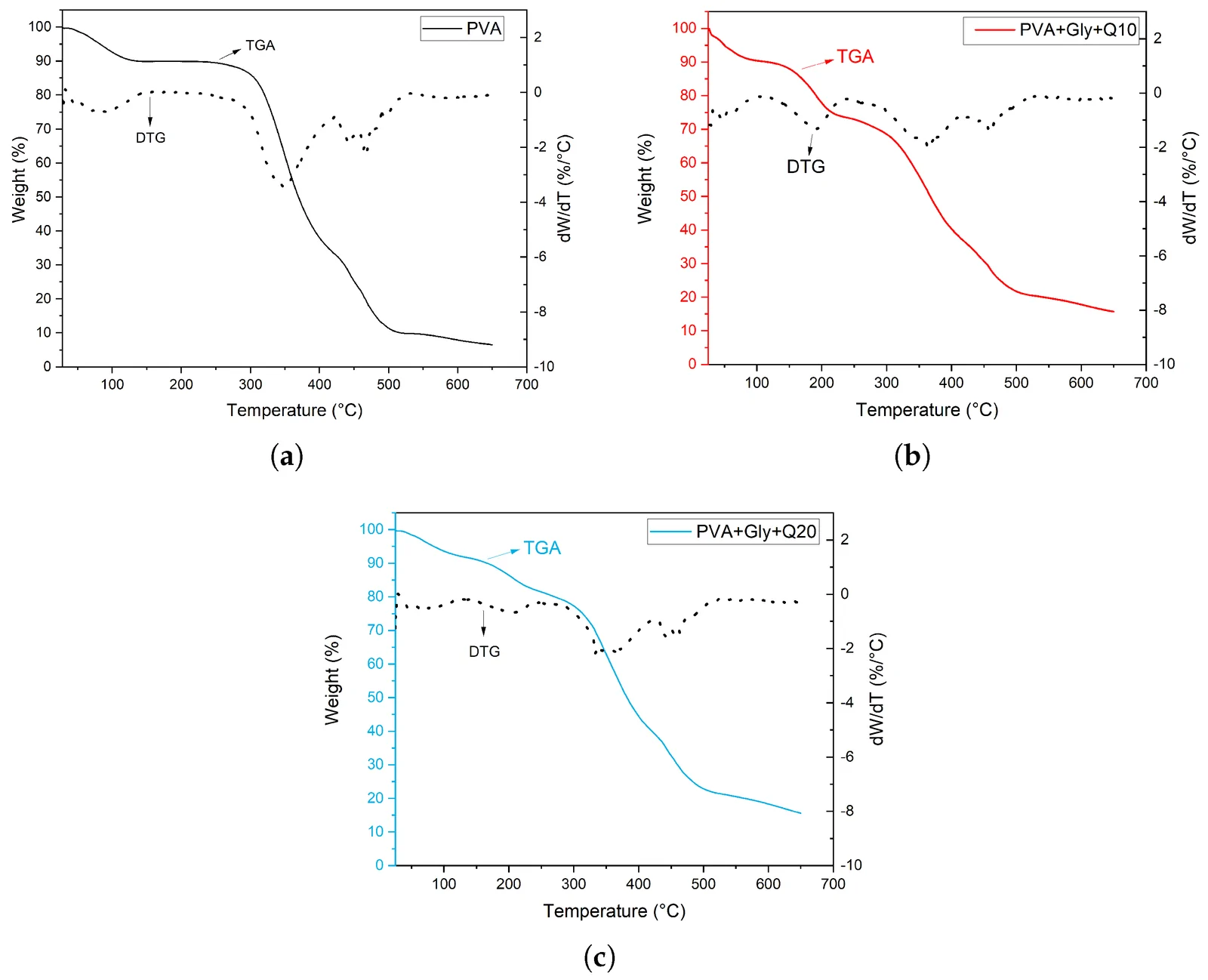
Thermogravimetric (TGA, solid lines) and derivative thermogravimetric (DTG, dotted lines) curves of (**a**) PVA, (**b**) PVA+Gly+Q10, and (**c**) PVA+Gly+Q20 films obtained under a nitrogen atmosphere. Characteristic thermal parameters derived from these curves are summarized in Table 2.

The TGA and DTG curves of PVA, PVA+Gly+Q10, and PVA+Gly+Q20 are presented in Figure 5. All formulations exhibited multistep mass-loss profiles, although the relative magnitude and temperature range of the events depended on the glycerol/quercetin ratio.

**Table 2:** Characteristic thermal parameters obtained from the TGA/DTG curves of the PVA-based films. $T_{5\%}$ and $T_{10\%}$ correspond to temperatures at 5% and 10% mass loss, respectively; $T_{\mathrm{onset}}$ is the extrapolated onset temperature of the principal degradation step; $T_{\max,\mathrm{DTG}}$ is the temperature of maximum mass-loss rate; and $R_{650}$ is the residual mass at 650 °C.

Sample	$T_{5\%}$ (°C)	$T_{10\%}$ (°C)	$T_{\mathrm{onset}}$ (°C)	$T_{\max,\mathrm{DTG}}$ (°C)	$R_{650}$ (%)
PVA	84	231	314	347	6.7
PVA+Gly+Q10	52	125	318	364	16.0
PVA+Gly+Q20	86	169	318	355	15.8

The early mass-loss behavior differed among the formulations. PVA+Gly+Q10 showed the lowest $T_{5\%}$ and $T_{10\%}$ values, whereas PVA+Gly+Q20 delayed these initial losses relative to PVA+Gly+Q10. These low-temperature events include the release of physically adsorbed water and other volatile species associated with the hydroxyl-rich matrices [22]. The earlier mass loss of PVA+Gly+Q10 is consistent with its greater glycerol fraction and with the lower-temperature mass-loss contributions reported for glycerol-containing PVA systems [13].

In contrast, the onset of the principal degradation step remained similar among the three films, with $T_{\mathrm{onset}}$ values between 314 and 318 °C. The $T_{\max,\mathrm{DTG}}$ values ranged from 347 to 364 °C, with the highest value observed for PVA+Gly+Q10. These results indicate that the additives affected the temperature of maximum mass-loss rate more noticeably than the onset of the principal degradation process.

The principal degradation event of PVA is associated primarily with dehydration and elimination reactions involving the PVA side groups and the consequent formation of unsaturated sequences. The subsequent mass loss at higher temperatures reflects continued chain scission and decomposition of the structures formed during the preceding stage [13].

The broader degradation profiles of PVA+Gly+Q10 and PVA+Gly+Q20 indicate that decomposition of the PVA-rich phase overlapped with processes involving glycerol, quercetin, and the molecular environments produced by these additives. The incorporation of glycerol and quercetin therefore redistributed mass loss over a broader temperature interval rather than uniformly shifting the degradation process toward higher temperatures. Comparable overlapping degradation events have been reported in PVA films containing glycerol or phenolic compounds [13,19].

The residual mass at 650 °C increased from 6.7% for PVA to 16.0% and 15.8% for PVA+Gly+Q10 and PVA+Gly+Q20, respectively. The similar residues of the two additive-containing films show that increasing the quercetin fraction from 10 to 20 wt.% did not produce a corresponding increase in the final residue. The higher residual masses relative to neat PVA are consistent with greater formation or retention of thermally persistent carbonaceous material under nitrogen.

### 3.6. Tensile Mechanical Properties

**Figure 6:**
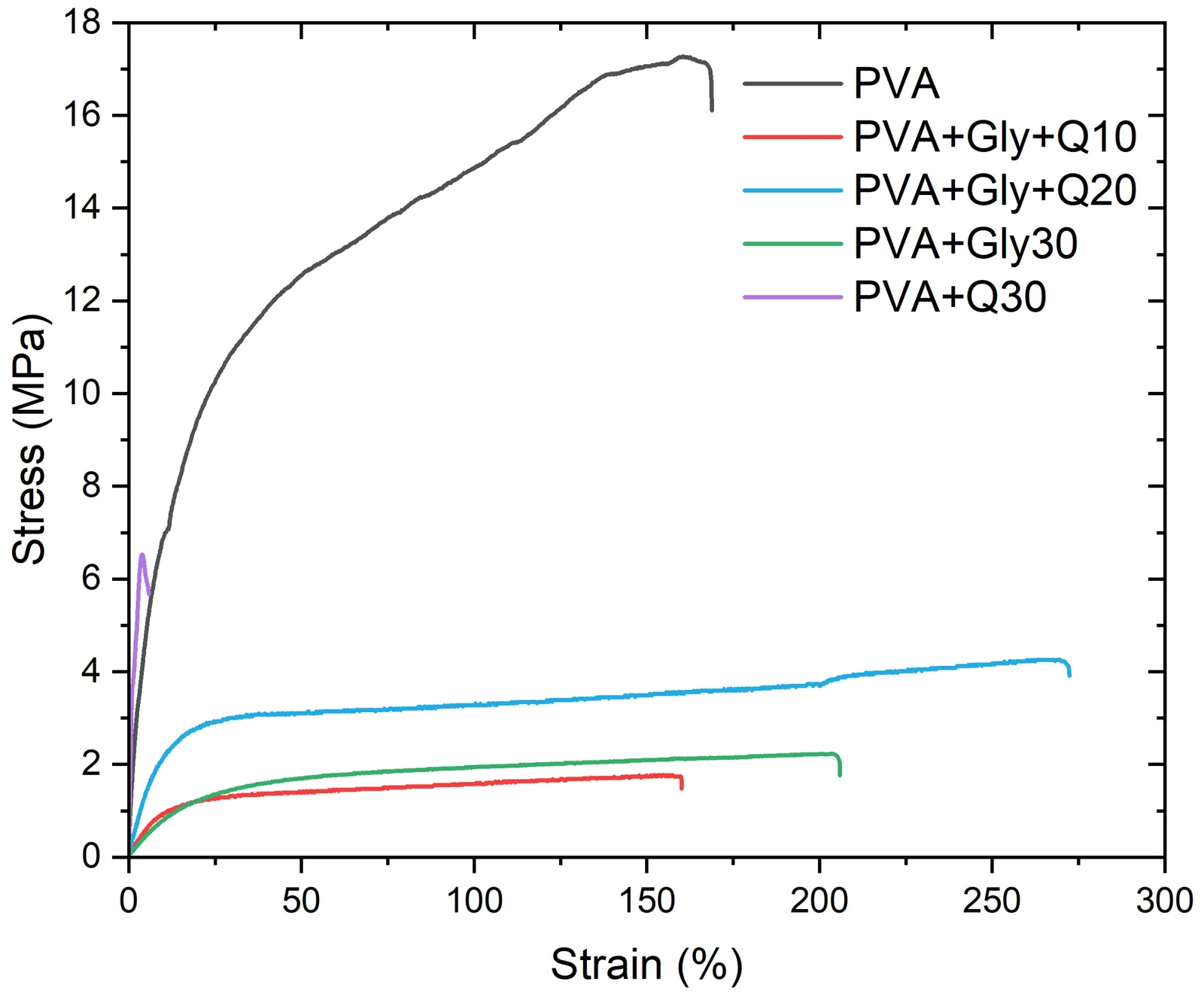
Representative engineering stress–strain curves of PVA, PVA+Gly30, PVA+Gly+Q10, PVA+Gly+Q20, and PVA+Q30 films tested at 22 °C. One representative valid specimen from each formulation is shown. PVA+Gly30 and PVA+Q30 were included as endpoint mechanical controls and were not part of the remaining physicochemical characterization.

Representative engineering stress–strain curves are shown in Figure 6, and the corresponding tensile parameters are summarized in Table 3. PVA+Gly30 and PVA+Q30 are included only in this mechanical section as endpoint controls for the glycerol-to-quercetin substitution series.

Neat PVA exhibited the highest maximum stress, 15.67±1.49 MPa, and differed significantly from all additive-containing formulations. PVA+Q30 showed the second-highest maximum stress, 6.55±0.61 MPa, and remained significantly stronger than PVA+Gly30, PVA+Gly+Q10, and PVA+Gly+Q20. The glycerol-containing formulations with 70 wt.% PVA did not differ significantly from one another in maximum stress, although PVA+Gly+Q20 showed the highest mean value within this group.

The endpoint controls revealed a pronounced shift in deformability across the substitution series. PVA+Gly30 reached a strain at break of 206.92±16.34%, whereas PVA+Q30 exhibited a much lower value of 12.53±8.75%. PVA+Gly+Q10 showed an intermediate mean strain at break of 132.23±30.54%. PVA+Gly+Q20 had the highest mean strain at break, 256.87±115.68%, but also showed substantial specimen-to-specimen variability. Consequently, its strain-at-break value was not statistically distinguishable from several of the other formulations under the Games–Howell comparison.

The apparent tensile modulus showed the clearest compositional contrast. PVA+Q30 exhibited a markedly higher modulus (404.69±45.72 MPa) than all other formulations, consistent with the steep initial slope and early rupture observed in Figure 6. In contrast, PVA+Gly30 and PVA+Gly+Q10 showed low apparent moduli of 8.70±0.68 and 13.56±3.43 MPa, respectively. PVA+Gly+Q20 exhibited an intermediate modulus of 26.61±3.40 MPa, which was significantly higher than those of PVA+Gly30 and PVA+Gly+Q10 but was not statistically different from neat PVA under the Games–Howell test.

The endpoint controls define two distinct mechanical limits for the glycerol-to-quercetin substitution series. PVA+Gly30 exhibited the lowest apparent tensile modulus (8.70±0.68 MPa) together with high extensibility, consistent with a strongly plasticized response. In contrast, PVA+Q30 showed a markedly higher apparent tensile modulus (404.69±45.72 MPa) and very low strain at break (12.53±8.75%), indicating a much more rigid and less deformable material. The ternary formulations progressively occupied the mechanical space between these two endpoints. In particular, the apparent tensile modulus increased from 8.70±0.68 MPa for PVA+Gly30 to 13.56±3.43 MPa for PVA+Gly+Q10 and 26.61±3.40 MPa for PVA+Gly+Q20 as glycerol was progressively replaced by quercetin. This trend provides the clearest evidence that the glycerol-to-quercetin balance shifts the films from a more compliant toward a more rigid mechanical response.

This evolution is physically consistent with the different roles of the two additives in the PVA matrix. Glycerol acts as a plasticizing component and is therefore associated with greater chain mobility and lower resistance to deformation, whereas increasing the quercetin fraction is associated with progressive stiffening of the films. The mechanical response, however, was not a simple linear interpolation between the two endpoints. PVA+Gly+Q20 combined an intermediate apparent modulus with the highest mean strain at break, although the large standard deviation of this parameter indicates substantial specimen-to-specimen variability. Thus, the mixed glycerol/quercetin formulations appear to define intermediate and composition-dependent mechanical regimes rather than a monotonic transition in all tensile properties. In particular, the modulus followed the clearest compositional progression, whereas maximum stress and strain at break showed a more complex response. These results indicate that the glycerol/quercetin ratio can tune the stiffness–strength–extensibility balance of the PVA-based films, without requiring a direct mechanistic interpretation beyond that supported by the present mechanical measurements.

### 3.7. Total Phenolic Content and DPPH Radical-Scavenging Activity

**Figure 7:**
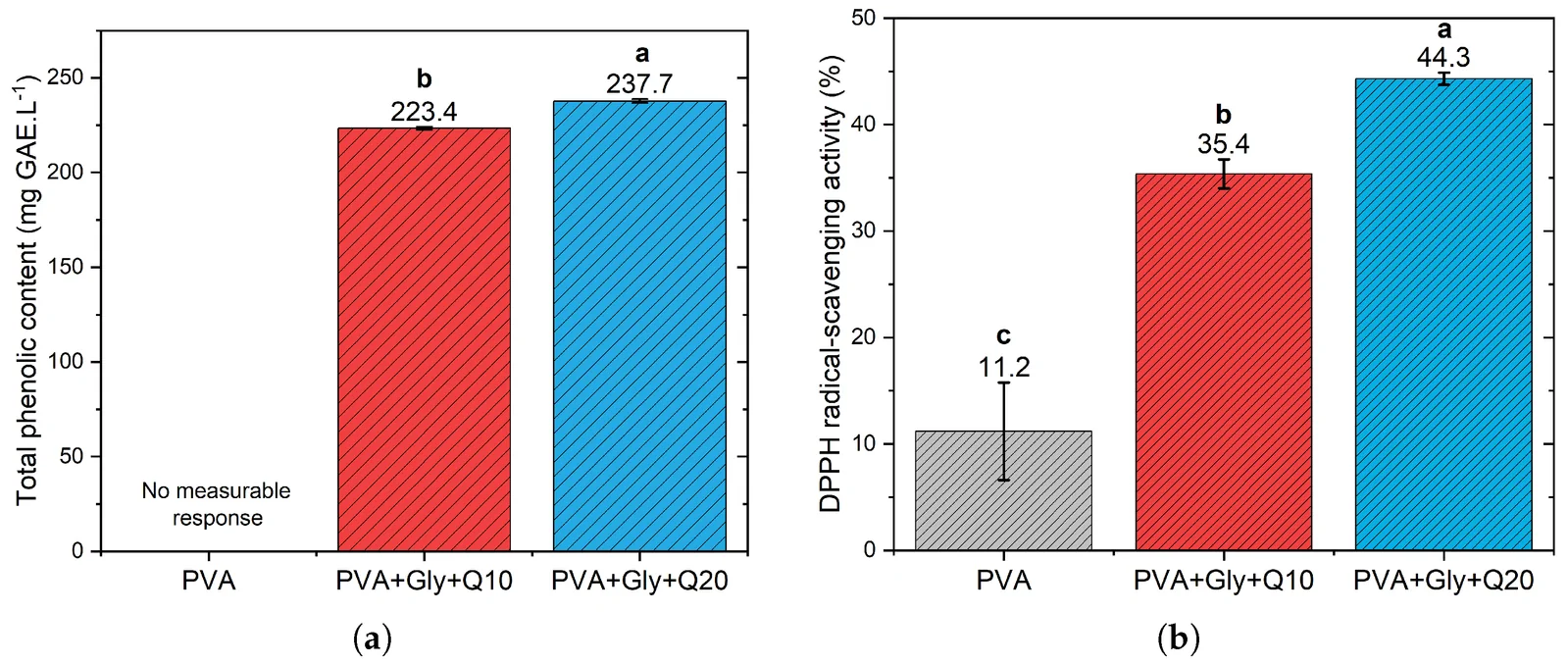
(**a**) Total phenolic content and (**b**) DPPH radical-scavenging activity of extracts from PVA, PVA+Gly+Q10, and PVA+Gly+Q20 films. Total phenolic content is expressed as milligrams of gallic acid equivalents (GAE) per liter of extract. Results are presented as mean ± standard deviation. Different letters indicate significant differences within each panel (p<0.05). In (**a**), PVA+Gly+Q10 and PVA+Gly+Q20 were compared using Welch’s *t*-test; neat PVA yielded zero absorbance and was excluded from the concentration-based comparison. In (**b**), the three formulations were compared using the Games–Howell test following Welch’s one-way ANOVA.

The total phenolic contents of the film extracts are presented in Figure 7a. PVA+Gly+Q10 and PVA+Gly+Q20 exhibited values of 223.35±0.26 and 237.74±1.39 mg GAE L^−1^, respectively. Partial replacement of glycerol by quercetin was associated with a 6.4% increase in mean total phenolic content, with a significant difference between the two formulations (Welch’s *t*-test, p=0.0023). Neat PVA yielded no measurable response under the assay conditions and was excluded from the concentration-based comparison.

The DPPH radical-scavenging activities of the PVA, PVA+Gly+Q10, and PVA+Gly+Q20 films are presented in Figure 7b. Neat PVA showed a comparatively low DPPH radical-scavenging activity of 11.18±4.58%, whereas PVA+Gly+Q10 and PVA+Gly+Q20 exhibited activities of 35.38±1.35% and 44.31±0.57%, respectively. The formulation effect was significant (Welch’s one-way ANOVA, p=0.0015), and all three formulations differed significantly from one another (Games–Howell test, p<0.05).

The low value obtained for neat PVA indicates that the polymer matrix has limited ability to neutralize DPPH radicals under the conditions employed. Although PVA contains numerous hydroxyl groups, these non-phenolic hydroxyl groups do not provide the same radical-scavenging response as the phenolic structure of quercetin.

The higher activity of the quercetin-containing films is primarily attributed to the phenolic hydroxyl groups of quercetin, which can donate hydrogen atoms or electrons to DPPH radicals. The resulting quercetin-derived radicals are stabilized by resonance within the conjugated aromatic structure. The higher total phenolic content and DPPH activity of PVA+Gly+Q20 follow a consistent formulation-dependent trend, complementing the ATR-FTIR contributions near 1600 and 1500 cm^−1^ assigned to quercetin.

The enhanced antioxidant activity is consistent with the findings of Vinhal et al. [20] for quercetin-containing gelatin/PVA films and Wang et al. [7] for PVA-based packaging materials incorporating quercetin-functionalized components.

Within the present formulation set, the higher activity of PVA+Gly+Q20 accompanied the partial replacement of glycerol by quercetin at a constant PVA fraction. This response therefore reflects the glycerol/quercetin balance and demonstrates that antioxidant functionality remained accessible in the extracts obtained from the dried films.

### 3.8. Water Vapor Permeability

Partial replacement of glycerol by quercetin was associated with a marked decrease in the mean water vapor permeability (WVP) of the ternary films (Table 4). PVA+Gly+Q10 exhibited the highest mean WVP, 29.90±11.74 g mm m^−2^ h^−1^ kPa^−1^, approximately four times the value obtained for neat PVA (7.24±2.36 g mm m^−2^ h^−1^ kPa^−1^). Increasing the quercetin-to-glycerol ratio in PVA+Gly+Q20 reduced the mean WVP by approximately 68% relative to PVA+Gly+Q10, yielding 9.48±7.34 g mm m^−2^ h^−1^ kPa^−1^. Owing to the relatively high dispersion of the measurements, however, Welch’s one-way ANOVA did not detect a significant formulation effect ($F_{\mathrm{Welch}}\left(2,3.01\right)=4.43$, p=0.127). The WVP results are therefore interpreted as formulation-dependent trends in the mean values rather than statistically established differences.

Water vapor transport involves water sorption into the film followed by diffusion through the polymer matrix. The greater glycerol fraction in PVA+Gly+Q10 can favor both processes: its affinity for water promotes sorption, while its plasticizing action increases chain mobility and facilitates diffusion. This interpretation is consistent with the more extensive wetting of PVA+Gly+Q10, whose apparent water contact angle reached approximately 20° at 60 s, compared with 35° for PVA+Gly+Q20. Reducing the glycerol fraction from 20 to 10 wt.% while increasing quercetin was thus accompanied by both less extensive wetting and a lower mean WVP.

The comparable apparent crystallinity indices of PVA+Gly+Q10 and PVA+Gly+Q20 (11.1% and 10.6%, respectively) indicate that the large difference in their mean WVP values is not accompanied by a meaningful difference in apparent crystallinity. The observed WVP trend is therefore more consistent with composition-dependent differences in water affinity and transport through the polymer matrix. Because glycerol and quercetin varied simultaneously, their individual contributions to this behavior cannot be isolated from the present formulation set.

PVA+Gly+Q20 exhibited a mean WVP closer to that of neat PVA than to PVA+Gly+Q10, while also showing higher mean DPPH radical-scavenging activity. Among the two ternary formulations, PVA+Gly+Q20 therefore showed a more favorable mean-value balance between water vapor permeability and antioxidant activity, supporting its further evaluation for active-packaging applications.

## 4. Conclusions

The glycerol/quercetin ratio modified the structural organization, surface morphology, wetting behavior, thermal-degradation profile, tensile response, water-vapor barrier performance, and antioxidant activity of the PVA-based films. PVA+Gly+Q10 showed faster time-dependent wetting and greater mass loss between 150 and 250 °C, whereas partial replacement of glycerol by quercetin reduced both effects in PVA+Gly+Q20. Both additive-containing films produced greater residual masses than neat PVA at 650 °C, although neither exhibited a clear increase in the onset temperature of degradation.

Under the common tensile-test condition of 22 °C, the glycerol-rich and quercetin-rich endpoint controls defined markedly different mechanical limits for the substitution series. PVA+Gly30 exhibited a low apparent tensile modulus (8.70±0.68 MPa) together with high extensibility, whereas PVA+Q30 exhibited a markedly higher apparent tensile modulus (404.69±45.72 MPa) and very low strain at break. The ternary formulations occupied intermediate mechanical regimes. In particular, the apparent tensile modulus increased progressively from PVA+Gly30 to PVA+Gly+Q10 and PVA+Gly+Q20 as glycerol was replaced by quercetin, whereas maximum stress and strain at break showed a non-monotonic response. PVA+Gly+Q20 exhibited the highest mean strain at break, although this parameter showed substantial specimen-to-specimen variability.

Partial replacement of glycerol by quercetin also increased the antioxidant response of the film extracts. DPPH radical-scavenging activity increased from 11.18% for neat PVA to 35.38% for PVA+Gly+Q10 and 44.31% for PVA+Gly+Q20. PVA+Gly+Q20 also exhibited a lower mean water-vapor permeability than PVA+Gly+Q10, although the formulation effect was not statistically significant. Taken together, the results show that the glycerol/quercetin balance can be used to tune the stiffness–strength–extensibility profile of PVA-based films while increasing antioxidant functionality. PVA+Gly+Q20 remains a promising formulation for further active-packaging development, although oxygen permeability, water solubility and swelling, quercetin migration or release, and performance in food simulants should be evaluated before its suitability for food-contact applications can be established.

## Figures and Tables

**Table 1:** Composition of the PVA/glycerol/quercetin films. Percentages are expressed relative to the total dry mass, excluding processing water.

Sample	PVA	Glycerol	Quercetin	PVA	Glycerol	Quercetin
	(wt.%)	(wt.%)	(wt.%)	(g)	(g)	(g)
PVA	100	0	0	0.7854	0	0
PVA+Gly+Q10	70	20	10	0.5498	0.1571	0.0785
PVA+Gly+Q20	70	10	20	0.5498	0.0785	0.1571

**Table 3:** Tensile properties of the PVA-based films tested at 22 °C. Five specimens were tested for each formulation. Results are presented as mean ± standard deviation. Within each tensile-property column, values that do not share a lowercase letter differ significantly according to Games–Howell pairwise comparisons following Welch’s one-way ANOVA (p<0.05).

Formulation	Maximum Stress $\sigma_{\max}$ (MPa)	Strain at Break $\varepsilon_{b}$ (%)	Apparent Tensile Modulus (MPa)
PVA	15.67±1.49 ^a^	157.40±21.46 ^ab^	80.50±15.43 ^a^
PVA+Gly30	2.23±0.18 ^c^	206.92±16.34 ^a^	8.70±0.68 ^b^
PVA+Gly+Q10	1.98±0.44 ^c^	132.23±30.54 ^b^	13.56±3.43 ^b^
PVA+Gly+Q20	3.88±0.98 ^c^	256.87±115.68 ^abc^	26.61±3.40 ^a^
PVA+Q30	6.55±0.61 ^b^	12.53±8.75 ^c^	404.69±45.72 ^c^

**Table 4:** Water vapor permeability (WVP) of the PVA-based films. Results are expressed as mean ± standard deviation (n=3).

Sample	WVP (g mm m−2 h−1 kPa−1)
PVA	7.24±2.36
PVA+Gly+Q10	29.90±11.74
PVA+Gly+Q20	9.48±7.34

## Data Availability

The datasets generated during and/or analyzed during the current study are available from the corresponding authors on reasonable request.

## Supplementary Materials

The following supporting information can be downloaded at: https://www.mdpi.com/article/10.3390/polym18192452/s1, Figure S1: XRD deconvolution of PVA, PVA+Gly+Q10, and PVA+Gly+Q20; Table S1: XRD deconvolution parameters, apparent crystallinity indices, sensitivity ranges, and goodness-of-fit results.
