# Supplementary material for: Effects of the Glycerol/Quercetin Ratio on the Structure and Tensile Behavior of PVA-Based Films
Source: Polymers (Basel). 2026 Oct 8;18(19):2452. doi: 10.3390/polym18192452 (PMC13666965; doi:10.3390/polym18192452)
Supplement: Supplementary file 1 [file polymers-18-02452-s001.zip › polymers-4578217-supplementary.pdf]

Supporting Information

Effects of the Glycerol/Quercetin Ratio on the Structure and Tensile Behavior of PVA-Based Films

Sarah Kalli Silva da Silva [1], Nathalia Vieira Villar de Nunes [1], Chiara das Dores do Nascimento [1,2], Eduarda Vieira Silva [1], Everton Granemann Souza [3,*], Camila Monteiro Cholant [1], Ivandra Ignes de Santi [1], Amanda Dantas de Oliveira [1], Cesar Aguzzoli [4], Alexandre Ferreira Galio [5] and André Luiz Missio [1,*]

[1] Graduate Program in Materials Science and Engineering (PPGCEM), Federal University of Pelotas (UFPel), Pelotas 96010-610, RS, Brazil

[2] Graduate Program in Sciences and Technologies in Education (PPGCITED), Federal Institute of Education, Science and Technology of Rio Grande do Sul (IFSul), Pelotas 96060-290, RS, Brazil

[3] Graduate Program in Electronics and Computer Engineering (MEEC), Catholic University of Pelotas (UCPel), Pelotas 96015-560, RS, Brazil

[4] Graduate Program in Materials Engineering and Science (PPGMAT), University of Caxias do Sul (UCS), Caxias do Sul 95070-560, RS, Brazil

[5] Graduate Program in Materials Science and Engineering (PPCEM), Federal University of Pampa (UNIPAMPA), Bagé 96413-172, RS, Brazil

* Correspondence: everton.granemann@ucpel.edu.br (E.G.S.); andre.missio@ufpel.edu.br (A.L.M.)

Additional XRD Analysis

X-ray diffraction (XRD) profiles were deconvoluted according to the procedure described in the main text, using the same sample designations defined therein. Two broad diffuse contributions (A1 and A2) were used to model scattering associated predominantly with the amorphous fraction, whereas the narrower contributions C1, C2, and C3 represented more ordered contributions associated with the crystalline fraction of the PVA matrix. The positions and widths of C1–C3 were constrained to common values across the three formulations, while their intensities and the parameters of A1 and A2 were independently optimized for each diffraction profile.

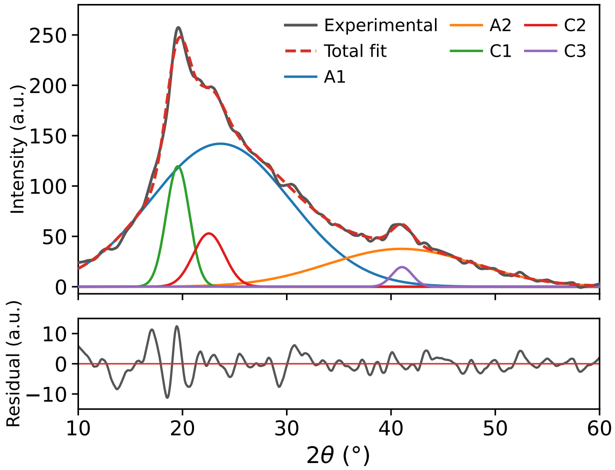

(a) PVA

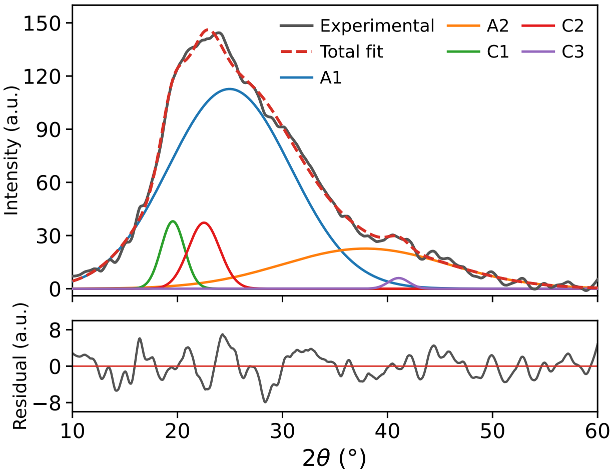

(b) PVA+Gly+Q10

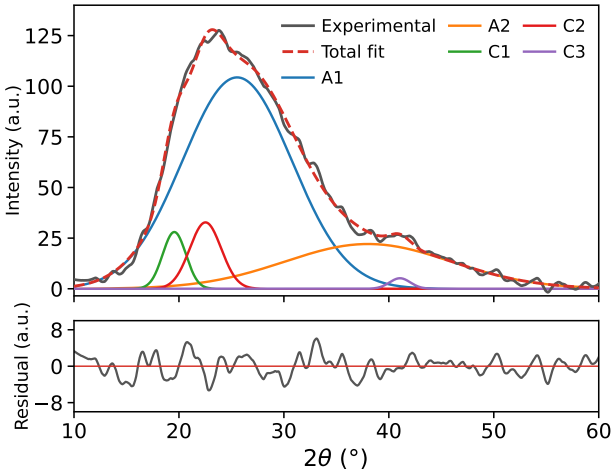

(c) PVA+Gly+Q20

Figure S1: XRD deconvolution of (a) PVA, (b) PVA+Gly+Q10, and (c) PVA+Gly+Q20. The experimental profiles correspond to the measured XRD signals after subtraction of the fitted baseline. The total fit represents the reconstructed diffraction profile obtained from the broad diffuse contributions A1 and A2, associated predominantly with the amorphous fraction, and the narrower ordered contributions C1–C3, associated with the crystalline fraction. The lower panels show the corresponding fitting residuals in arbitrary units (a.u.).

Table S1: Parameters obtained from the XRD deconvolution and corresponding apparent crystallinity and goodness-of-fit results. Panel (a) presents the broad diffuse contributions A1 and A2, associated predominantly with the amorphous fraction; panel (b) presents the narrower ordered contributions C1, C2, and C3, associated with the crystalline fraction; and panel (c) summarizes the apparent crystallinity index (X_c) and fitting quality. Here, μ denotes the fitted center position, FWHM the full width at half maximum, R^2 the coefficient of determination, and RMSE the root mean square error. The ordered contributions C1–C3 were constrained to common center positions and widths for all formulations. The reported X_c values correspond to the reference fit using a maximum Gaussian standard deviation of $\sigma_{max} = 1.5°$ for the ordered contributions, while the sensitivity ranges were obtained by repeating the analysis with $\sigma_{max} = 1.3°$ and 1.7° to assess the dependence of X_c on this fitting constraint.

(a) Broad diffuse contributions

Sample	μ_{A1} (°)	$FWHM_{A1}$ (°)	μ_{A2} (°)	$FWHM_{A2}$ (°)
PVA	23.64	15.81	41.01	16.51
PVA+Gly+Q10	24.96	13.74	37.90	18.62
PVA+Gly+Q20	25.54	12.26	38.02	18.70

(b) Ordered contributions

Component	μ (°)	FWHM (°)
C1	19.56	2.61
C2	22.53	3.53
C3	41.06	2.33

(c) Apparent crystallinity and fitting quality

Sample	X_c (%)	Sensitivity range (%)	R^2	RMSE (a.u.)
PVA	16.2	15.7–16.7	0.9975	3.32
PVA+Gly+Q10	11.1	10.2–12.0	0.9972	2.62
PVA+Gly+Q20	10.6	9.8–11.4	0.9976	2.13
